# Accuracy and reproducibility of CT right-to-left ventricular diameter measurement in patients with acute pulmonary embolism

**DOI:** 10.1371/journal.pone.0188862

**Published:** 2017-11-28

**Authors:** Yvonne M. Ende-Verhaar, Lucia J. M. Kroft, Inge C. M. Mos, Menno V. Huisman, Frederikus A. Klok

**Affiliations:** 1 Department of Thrombosis and Hemostasis, Leiden University Medical Center, Leiden, The Netherlands; 2 Department of Radiology, Leiden University Medical Center, Leiden, The Netherlands; Universite de Bretagne Occidentale, FRANCE

## Abstract

**Background:**

Right ventricular (RV) dysfunction caused by acute pulmonary embolism (PE) is associated with poor short- and long-term prognosis. RV dilatation as a proxy for RV dysfunction can be assessed by calculating the right-to-left ventricle diameter (RV/LV) ratio on standard computed tomography pulmonary angiography (CTPA) images. It is unknown whether dedicated training is required to accurately and reproducibly measure RV/LV ratio therefore we aimed to assess these parameters in residents in internal medicine without experience in CTPA reading.

**Methods:**

CTPA images of 100 patients with PE were assessed by three residents after single instruction, and one experienced thoracic radiologist. Maximum diameters were evaluated in the axial view by measuring the distance between the ventricular endocardium and the interventricular septum, perpendicular to the long axis of the heart. RV dilatation was defined as a ratio of ≥1.0. Interobserver accuracy and reproducibility was determined using Kappa statistics, Bland-Altman analysis and Spearman's rank correlation.

**Results:**

The kappa statistic for the presence of RV dilatation of the residents compared to the experienced radiologist ranged from 0.83–0.94. The average interobserver difference in calculated RV/LV ratio’s (±SD) between the three residents was: -0.01 (SD0.11), 0.07 (SD0.14) and 0.06 (SD0.18) with an overall mean RV/LV diameter ratio of 1.04. In line with this, Spearman's rank correlation coefficients were 0.92, 0.88 and 0.85 respectively indicating very good correlation (p<0.01 for all).

**Conclusion:**

After simple instruction, RV/LV diameter ratio assessment on CTPA images by clinical residents is accurate and reproducible, which is of help in identifying PE patients at risk.

## Introduction

Right ventricular (RV) dysfunction caused by acute pulmonary embolism (PE) is associated with poor short and long-term prognosis, i.e. higher risk of PE related mortality and chronic thromboembolic pulmonary hypertension (CTEPH) [1–3]. Several methods to determine RV dysfunction have been proposed and validated [4, 5]. RV dilatation based on right-to-left ventricle (RV/LV) diameter ratio on computed tomographic pulmonary angiography (CTPA) as a measure of RV dysfunction correlates well with echocardiographic parameters [6–8]. RV dilatation on CTPA has been shown to predict a higher 30-day mortality risk (OR 2.08; 95% confidence interval (CI) 1.63–2.66) in 4661 patients presenting with PE and even in 2254 haemodynamically stable patients (OR 1.64; 95%CI 1.06–2.52) [9]. The advantage of RV/LV diameter ratio measurement on CTPA compared to echocardiography is that it obviates the need of a second imaging test in addition to the diagnostic test applied to confirm the PE diagnosis.

International guidelines do not recommend standard RV/LV diameter ratio measurement in all patients with acute PE, although the initial risk assessment of PE also involves the measurement of RV function [1, 10]. Specifically, the presence of RV dysfunction as well as of biomarkers of cardiac overload and ischemia help differentiating between patients at intermediate-low risk of adverse outcome and patients at intermediate-high risk. The latter is an indication for close hemodynamic monitoring due to the 5.6% risk of hemodynamic deterioration in the first days after diagnosis [11]. RV/LV diameter ratio assessment may thus be useful in day-to-day clinical practice and especially in circumstances that echocardiography is not readily available.

The inter- and intra-observer agreement and reproducibility of RV/LV diameter ratio measurement by trained radiologists is reported to be very good with a Cohen’s Kappa statistic ranging from 0.80 to 0.87 [12–15]. The accuracy and reproducibility of RV/LV diameter ratio measurements by non-radiologist clinicians without dedicated training and expertise in CT reading is unknown. We aimed to assess the accuracy and reproducibility of CTPA RV/LV diameter ratio measurement by three residents in internal medicine without prior dedicated training in CT reading.

## Methods

### Study population

This is a post hoc analysis of a previously published observational prospective outcome study aimed at assessing the incremental value of ventricular function measurement with ECG-synchronized cardiac CTPA scanning over standard CTPA measured RV/LV ratio for predicting the short term prognosis in patients with acute PE [16, 17]. Consecutive, normotensive patients with suspected acute PE, based on a likely clinical probability by the Wells rule and/or an abnormal D-dimer test, were eligible for inclusion. Patients with renal function impairment, age < 18 years, pregnancy or allergy to contrast were excluded. A total of 430 consecutive haemodynamic stable patients were included and underwent standard CTPA and ECG-synchronized cardiac CT scanning, of whom 113 (26%) were diagnosed with acute PE [16, 17]. For the current analysis, the first 100 consecutive patients with confirmed PE were selected. Institutional review board (IRB) approval was obtained and written informed consent provided by all patients for the original study. The IRB of the LUMC waived the need for informed consent for this post-hoc analysis.

### CTPA reading

**Fig 1:**
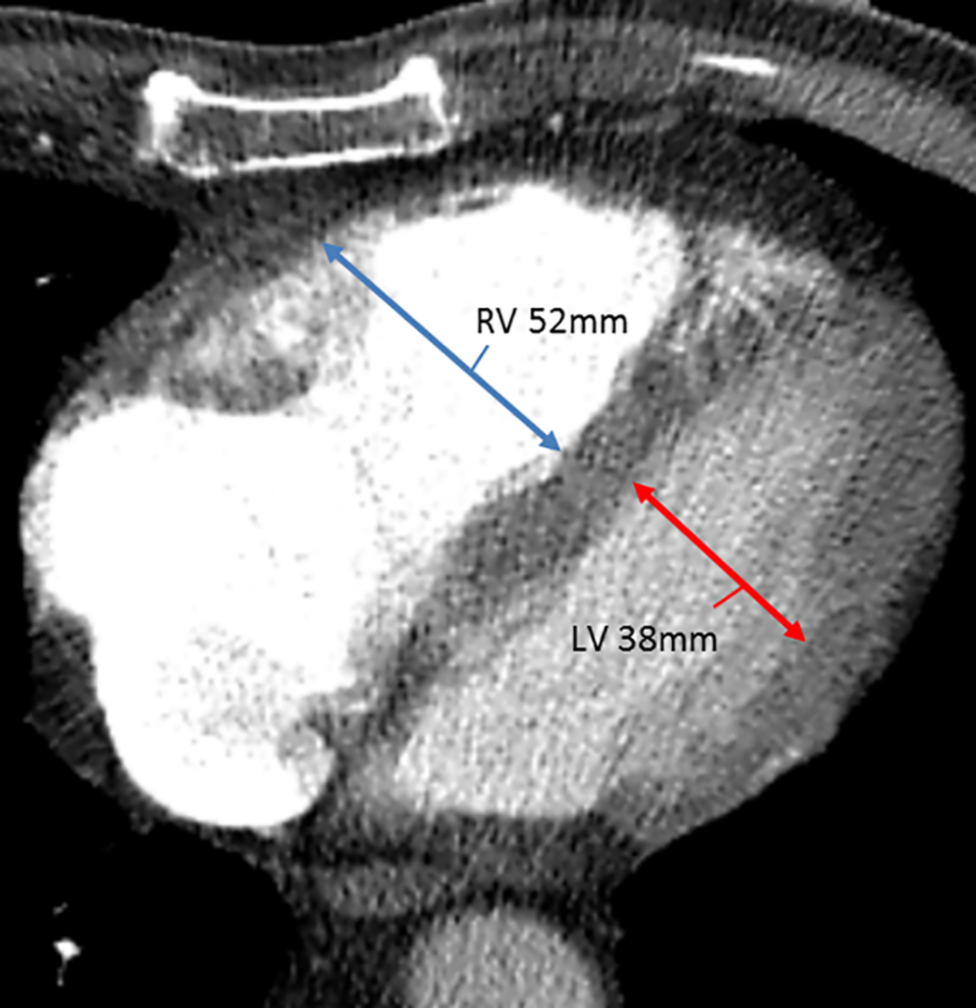
CTPA demonstrating the RV/LV ratio measurement. Note: CTPA: computed tomography pulmonary angiography; RV/LV: right-to left ventricle diameter ratio in this patient was 1.4.

The standard CTPA scans were reviewed chronologically by one expert thoracic radiologist (reviewer 1 (L.K)) with over 15 years of experience in pulmonary CTPA reading, two residents (reviewer 2 and 4 (Y.E-V and I.M)) and one senior resident with experience in VTE research (reviewer 3 (F.K.)), without specific training in CTPA reading. The experienced thoracic radiologist provided the following written instructions to the three residents: 1) evaluate the ventricle diameters in the standard axial view, 2) Measure the maximal distance between the ventricular endocardium and the interventricular septum, perpendicular to the long axis of the heart, and 3) Use the maximum dimensions for both ventricles which may be found at different levels [12, 18]. In addition one RV/LV diameter ratio measurement was demonstrated **(Fig 1).** All four reviewers were blinded to the findings of the other reviewers. RV dilatation was defined as a RV/LV diameter ratio of ≥1.0 [1].

### Study aim

The primary aim of the study was to evaluate the accuracy of assessing the presence or absence of RV dilatation, defined as an RV/LV diameter ratio of ≥1.0, by three residents in internal medicine without dedicated training in CT reading compared to the ruling of an experienced thoracic radiologist. The secondary aims of the study were to compare mean differences in the measured RV/LV diameter ratio in the individual study patients between the three residents internal medicine.

The primary endpoint was the kappa statistic for the presence or absence of RV dilatation measured by the three residents compared to the experienced thoracic radiologist. The secondary endpoint was the correlation coefficient between RV/LV ratio measurements among the three residents.

### Statistical analysis

Based on previous studies on this subject, we set our sample size at 100 CTPAs [12–15]. Baseline characteristics of the patients are provided with corresponding frequencies. Interobserver reproducibility for the dichotomous variable, i.e. a RV/LV diameter ratio of ≥ 1.0, of the thoracic radiologist compared to each of the three residents and among the residents was determined by using Cohen’s kappa-statistics. The kappa value for agreement was interpreted as follows: poor (< 0.20), fair (0.21–0.40), moderate (0.41–0.60), good (0.61–0.80) or very good (0.81–1.00) [19]. Further, Bland and Altman plots were used to represent the mean difference between the RV/LV diameter ratio measurements by the three residents [20]. We predefined adequate interobserver agreement on the Bland and Altman plot by a mean difference between 2 readers <0.1. Correlations between the measurements in individual patients were determined by Spearman's rank correlation. A correlation coefficient of 1 indicates a positive correlation while a coefficient of 0 represents no correlation. All analyses were performed using SPSS software version 23 for Windows IBM Corporation.

## Results

### Patients

One-hundred haemodynamically stable consecutive patients diagnosed with symptomatic acute PE were selected for the current analysis [16, 17]. Patient characteristics are provided in **Table 1**. Their mean age was 55 ± 16 years and 51 (51%) of the patients were male. Twenty-one patients (21%) had a history of venous thromboembolism, 38 patients (38%) had an unprovoked PE (bases on the absence of immobility, recent surgery, postpartum period or use of oral contraceptives or active malignancy). Twenty-four (24%) had an active malignancy.

**Table 1:** Patient characteristics.

	Patients (n = 100)
Age (years ± SD)	55 ± 16
Male sex (n,%)	51 (51%)
Previous PE/DVT (n,%)	21 (21%)
Immobility, surgery, trauma, postpartum, estrogen use (n,%)*	49 (49%)
Active malignancy (n,%)*	24 (24%)
Unprovoked PE (n,%)	38 (38%)
Inpatient (n,%)	17 (17%)
Left sided heart failure	3 (3%)

Note: PE: pulmonary embolism; DVT: deep vein thrombosis; n: number; SD: standard deviation

* 11 patients had an active malignancy and immobility, surgery, trauma, postpartum or estrogen use

### Accuracy of the RV/LV diameter ratio assessment

According to the measurement of the experienced radiologist, the RV was dilated (RV/LV diameter ratio of ≥1.0) in 42 CTPA scans, and the RV was not enlarged in 58 scans. Each resident individually measured the RV/LV diameter ratio of 93 (93%; 95%CI 86–97), 97 (97%; 95%CI 91–99) and 92 (92%; 95%CI 85–96) CTPA scans in accordance with the experienced radiologist resulting in a Cohen Kappa statistic of 0.86 (95%CI 0.75–0.96), 0.94 (95%CI 0.87–1.00) and 0.83 respectively (95%CI 0.72–0.94) **(Table 2).** The Cohen Kappa statistics between the residents internal medicine were 0.88 (95%CI 0.78–0.97; Reviewer 2 –Reviewer 3), 0.85 (95%CI 0.75–0.96; Reviewer 2 –Reviewer 4) and 0.85 (95%CI 0.75–0.96; Reviewer 3 –Reviewer 4). All discrepancies between the 3 residents concerned patients with RV/LV diameter ratio close to 1.0 **(S1 Table)**.

**Table 2:** Cohen kappa statistic of the experienced thoracic radiologist reviewer 1 and the three residents internal medicine reviewer 2–4.

Cohen kappa statistic	Kappa
Reviewer 1 –reviewer 2	0.86 (95%CI 0.75–0.96)
Reviewer 1 –reviewer 3	0.94 (95%CI 0.87–1.00)
Reviewer 1 –reviewer 4	0.83 (95%CI 0.72–0.94)
Reviewer 2 –reviewer 3	0.88 (95%CI 0.78–0.97)
Reviewer 2 –reviewer 4	0.85 (95%CI 0.75–0.96)
Reviewer 3 –reviewer 4	0.85 (95%CI 0.75–0.96)

Note: CI: confidence interval.

### Interobserver variability among the three residents internal medicine

**Fig 2:**
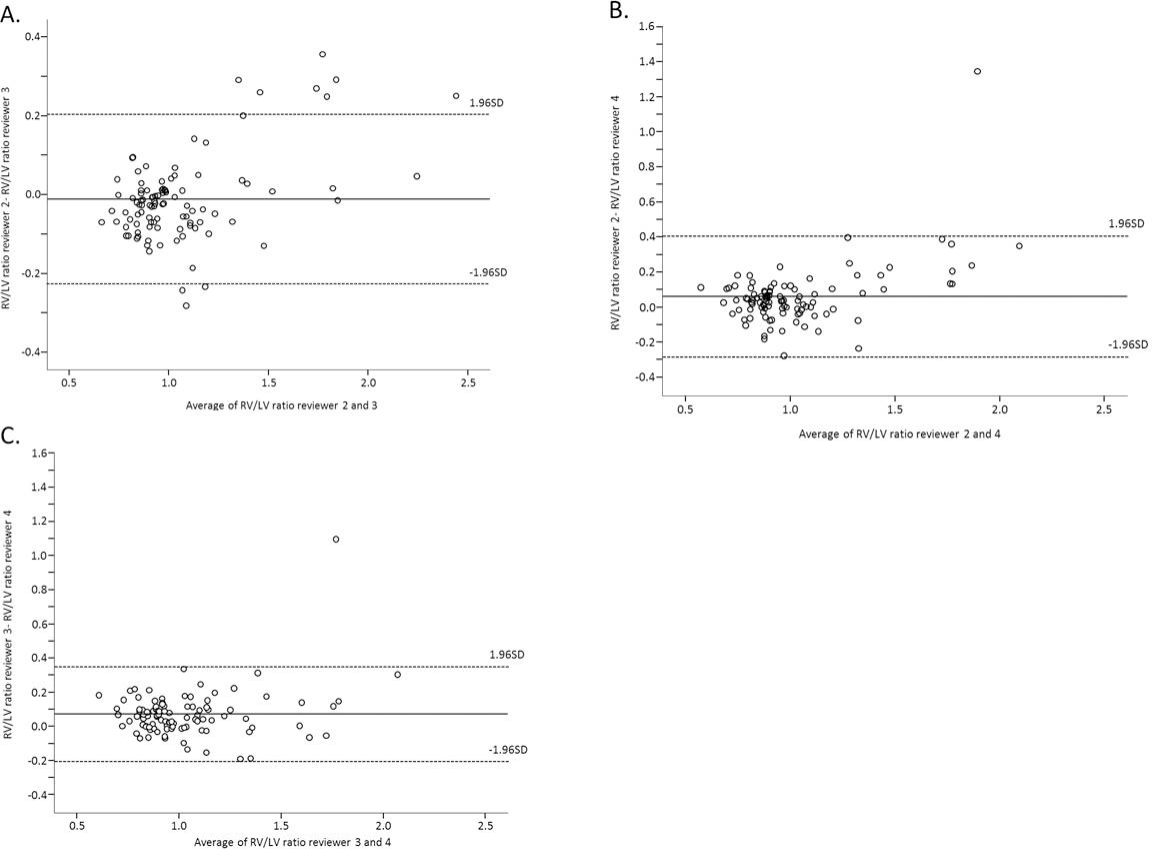
Bland and Altman analysis of the RV/LV diameter ratio measured by three residents internal medicine. Fig 2a reviewer 2 and 3, fig 2b reviewer 2 and 4, fig 2c reviewer 3 and 4. Note: RV/LV: right-to left ventricle diameter ratio.

The average RV/LV diameter ratio in the 100 measured CTPA scans by the three residents internal medicine was 1.06 (standard deviation(SD) 0.35), 1.07 (SD 0.29) and 1.00 (SD 0.26) respectively. On Bland Altman analysis, the mean difference in the calculated RV/LV diameter ratio’s (±SD) was -0.01 (SD0.11) (reviewer 2 and 3), 0.06 (SD0.18) (reviewer 2 and 4) and 0.07 (SD0.14) (reviewer 3 and 4) **(Fig 2)**. The Spearman's rank correlation coefficient was 0.92, 0.88 and 0.85 respectively (p<0.001 for all). The outlines in the Bland Altman plots were all in patients with RV/LV diameter ratio of larger than 1.5, i.e. those patients in whom RV overload is undoubtedly present. The differences in the RV/LV diameter ratio in these patients were mainly caused by variance in identification of the wall of the ventricular endocardium.

## Discussion

With the results of this study, we have shown that after a single focussed instruction, residents internal medicine without dedicated training and expertise in CT reading were able to accurately determine the presence or absence of RV dilatation as defined by a RV/LV diameter ratio of ≥ 1.0 on CTPA images in patients diagnosed with acute PE. Also, the mean difference in calculated RV/LV diameter ratio’s by the residents was very low (-0.01, 0.07 and 0.06 respectively), which was underlined by the very good correlation from the Spearman’s rank test (0.92, 0.88 and 0.85 respectively (p<0.01 for all)).

RV dilatation on CTPA is an indicator of RV dysfunction that can be useful in selecting PE patients with a high risk of an adverse short and long term outcome [1]. Even in haemodynamically stable patients, it has been clearly shown (OR of 1.64 (95%CI 1.06–2.52)) that an enlarged RV/LV diameter ratio on CTPA is associated with an increased risk of death at 30 days [9]. As for the long term prognosis, right ventricular dilatation at the moment of a PE diagnosis is an independent risk factor for CTEPH with a reported OR of 4.1 (95%CI 1.4–12) [2]. Alternative methods to assess RV dysfunction such as echocardiography are more time consuming, expensive and may not be available around the clock in all hospitals. With CTPA being the most commonly used method to diagnose acute PE, it is likely that this is the most simple and economic method to assess cardiac function at moment of diagnosis as well as post-hoc when patients visit the outpatient clinic for counselling on their long term prognosis.

Previous studies reported a good to very good inter- and intra-agreement on the CTPA RV/LV diameter ratio measurement between experienced radiologists **(Table 3)** using axial images. In four studies that evaluated a total of 393 patients with PE, the Cohen’s Kappa statistic for CT assessment of the presence of RV overload has ranged from 0.80 to 0.87 for trained radiologists with 5 to over 10 years of experience [12–15]. The first study retrospectively evaluated 61 unselected PE patients including 12 patients with massive PE and reported a kappa of at least 0.83 [13]. The remaining three studies were restricted to haemodynamic stable patients, with kappa statistics between 0.8 and 0.87 [12, 14, 15]. A fifth study evaluated the agreement between an experienced radiologist and a clinical physician with experience in CTPA reading for PE. In this study 460 unselected PE patients were included of which 49 were haemodynamically instable. The kappa statistic for a RV/LV diameter ratio of ≥0.9 was 0.88 [21].

**Table 3:** Studies evaluating the interobserver RV/LV diameter ratio agreement.

	Number of patients	Type of PE patients	Years of radiology experience	Kappa RV/LV ≥1/<1	Bland and Altman mean difference (SD)	Correlation coefficient
Jimenez *et al* 2012 [12]	96	Haemodynamically stable	Trained and certified radiologists	0.8	0.03 (0.23)	n.a.
Cok *et al* 2013 [13]	61	No selection	8 and 5 years	0.83–0.96*	n.a.	0.72–0.94*‡ (P<0.001)
Javadrashid *et al* 2015 [14]	63	haemodynamically stable and no pre-existing comorbidity	>10 years	0.87	n.a.	n.a.
Kang *et al* 2011 [15]	173	Haemodynamically stable	7 and 5 years	0.81	n.a.	0.89 (P<0.001) ¥
Kang *et al* 2010 [22]	50	No selection	6 and 3 years	n.a.	0.01 (0.20)	0.88 (P<0.001) ‡
Kumamaru *et al* 2012 [23]	30	No selection	Both 5 years	n.a.	n.a.	0.88 (P<0.001) ‡
Aribas *et al* 2014 [7]	120	Haemodynamically stable	5 and 12 years	n.a.	n.a.	0.85 (P<0.001) ‡
Ouriel *et al* 2017 [24]	10	RV/LV diameter ratio of ≥0.9	Experienced radiologist	n.a.	n.a.	0.98 (P<0.001) ‡
Becattini *et al*, 2011 [21]	260	No selection	Expert radiologist and a physician with experience on CTPA reading	0.88#	n.a.	0.91¥

Note: PE: pulmonary embolism; RV/LV: right-to left ventricle diameter ratio; SD: standard deviation; n.a.: not applicable

*different measurements including the RV/LV diameter ratio were mentioned within these numbers

# kappa based on a RV/LV ratio of ≥0.9 or <0.9

‡ Spearman rank correlation coefficient

¥ intra-class correlation coefficient

Two further studies assessed differences in the measured ratios using axial images. In the first study 96 haemodynamically stable patients were evaluated by trained and certified radiologists whose measurements of the RV/LV ratio differed only 0.03 (SD 0.23) on average [12]. The second study included 50 unselected PE patients, of whom 10 were haemodynamically instable, and found a mean difference of the measured ratios of 0.01 (SD 0.20) between 2 radiologists with 3 and 6 years of experience [22]. A final five studies covering a total of 444 PE patients whose CTPA images were read by radiologists with 3–12 years of experience, reported Spearman rank or intra-class correlation coefficients of 0.72 to 0.98 (P<0.001 for all), indicating a clear correlation between the measured ratio’s [13, 15, 22–24].

One earlier study described the interobserver agreement between radiologists and clinicians without specific training in chest CT reading [25]. This study described the interobserver agreement of the RV/LV diameter ratio on 113 CTPA scans of patients with suspected acute PE between two radiologists with 14 and 15 years of experience and inexperienced radiology residents [25]. The inter-reader variability as assessed by using interclass coefficients was 0.95.

To our knowledge, this study is the first study evaluating the accuracy and reproducibility of CTPA RV/LV diameter ratio measurement in PE patients by residents in internal medicine who did not have dedicated training or expertise in CT reading. This is relevant for daily clinical practice because in many cases clinical residents internal medicine, cardiology, pulmonology or emergency medicine are responsible for both the initial risk assessment and treatment as well as long term follow-up of patients with PE. This simple method is of help to the clinician in identifying the patient presenting with acute PE who is at higher risk of mortality in the acute moment [9] and on the long term of the development of CTEPH [2].

The main limitation of this study is that other signs of RV failure on CTPA such as enlargement of the pulmonary truncus and backflow of contrast in the vena cava were not studied but may be relevant as well in the evaluation of RV function on CTPA. Only haemodynamically stable patients were included making our results only applicable to that patient category. The RV/LV diameter ratio depends on the diameter of the LV as well. In patients with a pathologically enlarged LV, as was present in 3 patients of this current analysis, RV dilatation based on RV/LV diameter ratio could have been missed. This was a post hoc analysis of patients diagnosed with PE in our centre from an observational multicenter study. Therefore, a formal power analysis was not performed and the sample size was based on previous studies on this subject (**Table** 3). Also, three residents internal medicine were selected to perform all measurements. We did not formally prove that our results can be translated to residents from other hospitals or countries, or from other specialties (cardiology, pulmonology), although we would not expect relevant differences.

In conclusion, the presence of RV dilatation on CTPA in patients with acute PE were accurately assessed by clinical residents without dedicated training in CT reading but after simple instruction. This is of help in identifying PE patients at higher risk of short and long term adverse outcome.

## Supporting information

S1 Table Complete dataset. (SAV)
